# The Molecular and Cellular Mechanisms of Melatonin: From Physiological Actions to Clinical Applications in Reproductive Medicine

**DOI:** 10.3390/ijms27146524

**Published:** 2026-07-22

**Authors:** Kelly I-Rong Lee, Jie-Hong Chen, Kuo-Hu Chen

**Affiliations:** 1 School of Medicine, College of Medicine, MacKay Medical University, New Taipei City 25245, Taiwan; kellya05.kl@gmail.com (K.I.-R.L.); albertjhc@gmail.com (J.-H.C.); 2 Department of Obstetrics and Gynecology, Taipei Tzu-Chi Hospital, The Buddhist Tzu-Chi Medical Foundation, New Taipei City 23142, Taiwan; 3 School of Medicine, Tzu-Chi University, Hualien 97004, Taiwan

**Keywords:** melatonin, mitochondria, mitochondrial quality control, reproductive medicine

## Abstract

Melatonin has evolved from its classical role as a pineal-derived circadian regulator to a molecule increasingly recognized for its mitochondrial and cytoprotective functions. This review examines the molecular mechanisms and translational implications of melatonin within a mitochondria-centered framework, with particular emphasis on reproductive medicine. Available evidence suggests that melatonin may influence mitochondrial quality control (MQC) through multiple interconnected processes, including ROS regulation, mitochondrial dynamics, mitophagy, biogenesis, and mitochondrial inflammatory signaling. In mitochondria, melatonin can attenuate electron transport chain-derived oxidative stress through direct radical-scavenging reactions, antioxidant metabolite formation, and indirect activation of endogenous antioxidant systems. Experimental studies further suggest that melatonin may modulate Drp1-mediated fission, OPA1- and Mfn1/2-associated fusion, PINK1/Parkin-mediated mitophagy, and SIRT1/PGC-1α-related mitochondrial biogenesis. In reproductive medicine, melatonin has been investigated as a potential adjunctive strategy in assisted reproductive technology, endometriosis, and polycystic ovary syndrome. However, clinical evidence remains heterogeneous, and most human studies have evaluated reproductive or biochemical outcomes rather than direct MQC-related biomarkers. Therefore, although melatonin represents a promising mitochondria-targeted adjunct, standardized dosing strategies, tissue-level pharmacodynamic assessment, and validated mitochondrial biomarkers are needed to determine whether these mechanisms translate into reproducible clinical benefit.

## 1. Introduction

### 1.1. Evolution of the Melatonin Paradigm: From Circadian Regulator to Mitochondrial Guardian

Melatonin (N-acetyl-5-methoxytryptamine), first isolated from the bovine pineal gland in 1958, was originally characterized as a neurohormone regulating circadian rhythms and seasonal physiology [1,2]. For decades, its biological role was largely interpreted through its systemic secretion from the pineal gland under the control of the suprachiasmatic nucleus (SCN), coordinating sleep–wake cycles and photoperiodic adaptation [3]. This classical view has been expanded by the recognition of extrapineal melatonin synthesis in tissues such as the ovaries, placenta, gastrointestinal tract, and immune cells. Functionally, extrapineal melatonin appears to exert tissue-specific effects [1]. In the ovary, melatonin has been implicated in follicular redox regulation, oocyte maturation, and protection of granulosa cells against oxidative stress [4]. In the placenta, melatonin may contribute to maternal–fetal redox balance, trophoblast function, and protection against oxidative injury [5]. In the gastrointestinal tract, locally produced melatonin has been associated with mucosal protection, epithelial homeostasis, and regulation of immune–neuroendocrine interactions [2]. In immune cells, melatonin has been reported to modulate cytokine production, oxidative stress, and inflammatory signaling [2]. These tissue-specific actions support the concept that melatonin functions not only as a circulating neurohormone but also as a locally acting cytoprotective molecule [4,5,6].

A further conceptual advance has emerged from the identification of melatonin biosynthetic enzymes—arylalkylamine N-acetyltransferase (AANAT) and acetylserotonin O-methyltransferase (ASMT)—within mitochondria [7,8]. This finding suggests that melatonin synthesis is spatially coupled to subcellular environments characterized by high metabolic activity and oxidative stress [8]. Accumulating evidence suggests that melatonin may act through coordinated regulatory systems that are closely linked to mitochondrial homeostasis, rather than through a single isolated pathway [7,8,9]. Rather than diminishing its circadian role, intramitochondrial localization expands melatonin’s functional repertoire, supporting a model in which it may contribute to cellular homeostasis by helping maintain mitochondrial integrity under metabolic and oxidative stress [7,8].

### 1.2. The Clinical Imperative: Oxidative Stress and Mitochondrial Dysfunction in Reproductive Pathology

The relevance of a mitochondria-centered view of melatonin is particularly evident in reproductive medicine, where infertility affects approximately 10–15% of couples worldwide and ovarian aging remains a major limiting factor [4,10,11]. Declining oocyte competence is strongly associated with mitochondrial dysfunction, characterized by excessive reactive oxygen species (ROS) generation, impaired bioenergetics, and mitochondrial DNA (mtDNA) instability [4,12]. Similarly, endometriosis—affecting approximately 10% of reproductive-aged women—is increasingly discussed in relation to mitochondrial dysfunction, oxidative stress, and dysregulated inflammatory signaling within ectopic lesions [13,14,15].

Beyond reproductive disorders, mitochondrial oxidative dysfunction may also represent a convergent biological feature in other conditions in which melatonin has been investigated, including PCOS and immune-mediated hyperinflammatory states [16,17,18]. Despite substantial evidence implicating oxidative damage in these conditions, conventional antioxidant therapies have produced inconsistent clinical outcomes [4,5]. A key limitation of these interventions is their limited capacity to accumulate within mitochondria, where ROS generation primarily occurs [5,9]. These observations highlight not only clinical relevance but also underscore the suitability of these disease contexts as in vivo systems for investigating mitochondrial regulatory mechanisms under sustained oxidative and inflammatory stress.

### 1.3. A Mitochondria-Centered Mechanistic Framework

Unlike conventional antioxidants, melatonin exhibits amphiphilic and lipophilic properties that enable it to traverse all biological membranes and accumulate within the mitochondrial matrix [4,6,9]. This property provides a mechanistic basis for a mitochondria-centered framework in which melatonin’s diverse biological actions can be integrated through the regulation of mitochondrial quality control (MQC)—a collective term encompassing mitochondrial dynamics (fusion and fission), mitophagy, and mitochondrial biogenesis [6,7,9,19,20]. From a molecular systems perspective, MQC represents a higher-order regulatory architecture that integrates redox balance, bioenergetics, and inflammatory signaling at the subcellular level [19].

At the mitochondrial level, melatonin directly attenuates ROS generation at the electron transport chain, preserves mitochondrial bioenergetics critical for oocyte maturation and early embryonic development, and stabilizes mitochondrial DNA integrity [4,9,21]. Beyond direct redox regulation, melatonin modulates MQC processes by maintaining fusion–fission balance, promoting mitophagy, and suppressing ROS-dependent inflammatory signaling pathways such as NF-κB and NLRP3 inflammasome activation [19,22,23,24,25]. Through both receptor-dependent (MT1/MT2-mediated) and receptor-independent mechanisms, melatonin’s biological effects appear to converge on pathways involved in mitochondrial homeostasis and cellular stress resilience [7,9,19].

**Figure 1:**
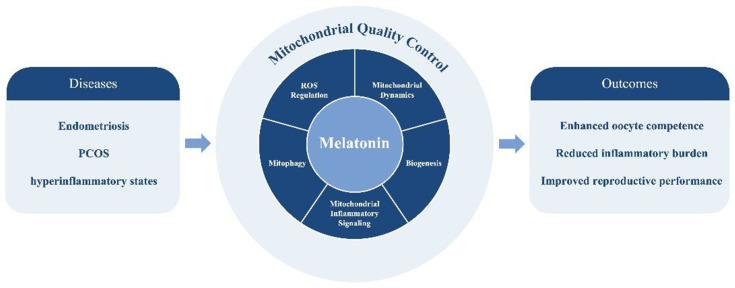
**Conceptual framework of melatonin-mediated mitochondrial quality control (MQC).** The diagram illustrates the systems-level conceptual framework proposed in this review. **Left panel (Diseases):** Target reproductive pathologies characterized by mitochondrial dysfunction, encompassing assisted reproductive technology (ART) contexts, endometriosis, polycystic ovary syndrome (PCOS), and hyperinflammatory states. **Center (MQC Hub):** Melatonin is positioned at the center of the MQC framework and is proposed to modulate five interconnected subcellular pillars: ROS regulation, mitochondrial dynamics, biogenesis, mitochondrial inflammatory signaling, and mitophagy. **Right panel (Outcomes):** By modulating this MQC network, melatonin may influence disease-relevant mitochondrial and inflammatory processes associated with oocyte competence, inflammatory burden, and reproductive outcomes.

Importantly, while numerous reviews have discussed melatonin’s antioxidant and mitochondrial effects, relatively few have framed its biological actions within the integrative architecture of mitochondrial quality control (MQC). We propose that MQC—encompassing mitochondrial dynamics, mitophagy, and biogenesis—may serve as a useful systems-level framework for interpreting melatonin’s diverse molecular actions and heterogeneous clinical observations. This conceptual distinction forms the central thesis of the present review. The integrative framework proposed in this review is illustrated in Figure 1, which conceptualizes melatonin as a system-level regulator of mitochondrial quality control (MQC). By positioning melatonin within five interconnected subcellular pillars, this framework may help explain how mitochondrial mechanisms contribute to disease-related phenotypes in reproductive and hyperinflammatory disorders, while highlighting the need for direct clinical validation.

### 1.4. Aim of the Review

Although melatonin has been extensively investigated across a wide range of biological contexts, an integrative synthesis linking its intramitochondrial mechanisms to clinically meaningful outcomes remains limited. Existing reviews have largely catalogued melatonin’s biological effects in a descriptive manner—emphasizing its antioxidant, anti-inflammatory, or cytoprotective properties as distinct functional categories. In contrast, the present review advances a unifying conceptual framework by positioning mitochondrial quality control (MQC) as the central organizing principle through which these seemingly independent actions converge. By reframing melatonin’s pleiotropic effects within the regulatory architecture of MQC, we aim to provide a system-level interpretation that connects molecular mechanisms with translational heterogeneity observed in clinical practice. With a primary emphasis on molecular and subcellular mechanisms, this narrative review aims to address this issue by examining melatonin through a mitochondria-centered framework, with particular emphasis on the role of mitochondrial quality control in reproductive disorders. We integrate current knowledge of melatonin biosynthesis, receptor-dependent and receptor-independent molecular pathways, and critically evaluate clinical evidence in assisted reproductive technology and endometriosis. In addition, we identify key translational limitations—including heterogeneity in dosing strategies and the limited incorporation of mitochondrial-specific biomarkers—that must be discussed to advance mechanistically informed and precision-based therapeutic approaches in reproductive medicine.

## 2. Methods of Literature Review

This narrative review was conducted to summarize experimental and clinical evidence regarding melatonin, mitochondrial function, and reproductive medicine. In the literature, articles published during 1997–2026 were considered. Relevant studies were identified through searches of PubMed and Ovid MEDLINE using combinations of the following terms: “melatonin”, “mitochondria”, “mitochondrial quality control”, “oxidative stress”, “reproductive medicine”, “assisted reproductive technology”, “endometriosis”, and “polycystic ovary syndrome”. In April 2026, we collected basic and clinical studies that investigated the cellular and molecular mechanisms of melatonin, as well as its physiological actions and clinical applications in reproductive medicine. In the second stage, only full-text articles published in English were considered. At the same time, duplicated articles were also excluded.

Studies were selected if they addressed melatonin biology, mitochondrial mechanisms, oxidative or inflammatory signaling, or clinical applications in reproductive disorders. In the third stage, articles with insufficient methodological detail and studies with unclear outcomes were excluded. Two reviewers in the field independently inspected the studies for relevance and scientific quality. Studies with poor research design, questionable methods or unclear outcomes were excluded to ensure the quality of retrieved studies. Finally, a total of 83 articles were eligible for inclusion in the current review. Because this was designed as a narrative rather than a systematic review, formal risk-of-bias assessment and quantitative meta-analysis were not performed.

## 3. Molecular Mechanisms: The Mitochondria-Centered Framework

### 3.1. Biosynthesis and Metabolism: The Shift Toward Intramitochondrial Production

Classically, melatonin biosynthesis was described as a circadian process occurring exclusively in pinealocytes, driven by rhythmic expression of arylalkylamine N-acetyltransferase (AANAT) under the control of the suprachiasmatic nucleus [26]. This viewpoint has been revised by evidence suggesting that melatonin synthesis is phylogenetically conserved and has been reported in mitochondria of diverse cell types [8,9].

Biochemically, tryptophan is converted to serotonin, which is subsequently acetylated and methylated to form melatonin [27]. Crucially, both the rate-limiting enzyme AANAT and the terminal enzyme ASMT have been localized within the mitochondrial matrix [7]. This subcellular compartmentalization enables on-site melatonin synthesis at locations characterized by high metabolic activity and oxidative burden [5,28]. As mitochondria represent the principal intracellular source of reactive oxygen species (ROS) generated during oxidative phosphorylation, local melatonin production establishes an automitocrine signaling loop, whereby mitochondria generate melatonin to counteract their own ROS output [7].

In contrast to pineal-derived melatonin, which is released into the circulation in a circadian-dependent manner, intramitochondrial melatonin synthesis appears largely independent of light–dark cues and instead responds dynamically to cellular metabolic demand [28,29]. This distinction highlights a functional dichotomy between systemic melatonin signaling and locally produced mitochondrial melatonin, the latter primarily serving cytoprotective and homeostatic roles [30,31].

### 3.2. Receptor-Dependent Signaling: Integration of Membrane and Nuclear Pathways with Mitochondrial Function

Melatonin exerts part of its biological activity through high-affinity, membrane-bound G protein-coupled receptors, MT1 and MT2 [1]. Upon ligand binding, these receptors predominantly couple to inhibitory Gi proteins, resulting in suppression of adenylate cyclase activity and reduced intracellular cyclic AMP levels [32]. Beyond their classical role in circadian regulation, MT1/MT2 signaling activates downstream pathways—including PI3K/Akt and MAPK/ERK—that influence mitochondrial survival and stress responses [33]. Through these signaling cascades, melatonin modulates mitochondrial membrane potential, inhibits pro-apoptotic mediators such as Bax, and reduces susceptibility to mitochondrial permeability transition pore opening [33,34]. These effects indirectly support mitochondrial integrity and bioenergetic stability, particularly under conditions of oxidative or inflammatory stress [18,33].

In addition to membrane receptors, melatonin interacts with nuclear retinoid-related orphan receptors (RORα/RZR), enabling transcriptional regulation of genes involved in antioxidant defense and circadian-metabolic coupling [35]. RORα activation directly regulates the transcription of core clock genes such as BMAL1, an effect further facilitated by SIRT1-dependent deacetylation of PGC-1α, which enhances RORα transcriptional activity [20]. Through modulation of circadian–metabolic gene programs, this pathway contributes indirectly to redox balance and mitochondrial function [20]. Importantly, receptor-dependent pathways appear to complement—but not fully account for—the extensive intramitochondrial actions of melatonin, implicating that receptor-independent mechanisms play an important role within the mitochondrial compartment [36,37].

### 3.3. Receptor-Independent Actions and Mitochondrial Quality Control

The distinctive therapeutic profile of melatonin in reproductive and inflammatory disorders is largely attributable to its receptor-independent actions within mitochondria [38]. Owing to its amphiphilic and lipophilic nature, melatonin readily traverses all biological membranes and accumulates within the mitochondrial matrix at concentrations far exceeding those in the cytosol or circulation [33]. This preferential mitochondrial accumulation positions melatonin to directly interact with redox-active sites and structural components of the mitochondrial network, enabling coordinated regulation of mitochondrial quality control (MQC) [19].

At the level of the electron transport chain (ETC), electron leakage from complexes I and III can promote the partial reduction of molecular oxygen to superoxide anion (O_2_•^−^) [21]. Superoxide is subsequently converted by mitochondrial superoxide dismutase 2 (SOD2) into hydrogen peroxide (H_2_O_2_), which can diffuse across mitochondrial compartments and, in the presence of transition metals, generate highly reactive hydroxyl radicals (•OH) through Fenton-type chemistry [12]. In addition to these ROS, mitochondrial oxidative stress may also generate reactive nitrogen species-related oxidants, including peroxynitrite (ONOO^−^), particularly when superoxide reacts with nitric oxide [37]. Thus, ETC-derived oxidative stress is not limited to superoxide but involves a cascade of reactive intermediates capable of damaging mitochondrial proteins, lipids, and mtDNA.

Melatonin may attenuate this oxidative cascade through both direct radical-scavenging reactions and indirect enhancement of mitochondrial antioxidant defenses [5]. Directly, melatonin can react with highly reactive species such as hydroxyl radicals (•OH), superoxide-derived radicals, peroxynitrite-related oxidants, and other oxygen-centered radicals [12]. During these reactions, melatonin undergoes oxidative modifications that generate metabolites including cyclic 3-hydroxymelatonin (C3-OHM), N1-acetyl-N2-formyl-5-methoxykynuramine (AFMK), and N1-acetyl-5-methoxykynuramine (AMK) [4]. Importantly, AFMK and AMK retain antioxidant and radical-scavenging capacity, thereby extending the antioxidant effect beyond the parent molecule. This sequential conversion has been proposed as a melatonin antioxidant cascade, in which melatonin and its metabolites collectively neutralize multiple reactive species [36,39].

Indirectly, melatonin has also been reported to support endogenous mitochondrial antioxidant systems [21]. For example, melatonin may enhance the SIRT3/SOD2 axis, improve electron transport chain efficiency, and reduce electron leakage from complexes I and III [8,28]. Through this combined direct and indirect mechanism, melatonin may reduce oxidative damage to mitochondrial proteins, membranes, and mtDNA, thereby supporting mitochondrial bioenergetic stability under oxidative stress [4,37].

Beyond redox control, melatonin has been shown in experimental models to modulate several components of mitochondrial quality control (MQC), including mitochondrial dynamics under oxidative stress conditions [33]. It suppresses pathological mitochondrial fission by inhibiting excessive activation and phosphorylation of dynamin-related protein 1 (Drp1), thereby preventing fragmentation and apoptosis under oxidative stress [40]. Reduced Drp1 activity preserves mitochondrial network continuity, maintains cristae organization, and prevents bioenergetic collapse [41]. Concurrently, melatonin supports mitochondrial fusion through preservation of optic atrophy protein 1 (OPA1), optimizing inner membrane architecture and respiratory efficiency [33,42].

Selective elimination of dysfunctional mitochondria via mitophagy represents a second critical component of mitochondrial quality control (MQC) [19,25]. The canonical PINK1/Parkin pathway is initiated when mitochondrial damage causes a loss of mitochondrial membrane potential (ΔΨm) [42]. Under this condition, PINK1 accumulates on the outer mitochondrial membrane instead of being imported and degraded [42]. Stabilized PINK1 then recruits and activates Parkin, an E3 ubiquitin ligase, which ubiquitinates outer mitochondrial membrane proteins and marks the damaged mitochondrion for autophagic recognition [43]. Ubiquitinated mitochondria are subsequently linked to LC3-positive autophagosomal membranes and degraded after autophagosome–lysosome fusion [25,44]. In selected experimental models, melatonin has been reported to support PINK1/Parkin-mediated mitophagy and autophagic flux [33,44]. This effect may be mediated, at least in part, by reducing mitochondrial ROS burden and by activating SIRT1-associated autophagic signaling [25]. By facilitating clearance of damaged mitochondria and coupling mitochondrial removal with biogenesis, melatonin may help maintain mitochondrial population quality [19,44]. Because PINK1/Parkin signaling can vary according to disease context and baseline mitophagy status, melatonin is best described as normalizing dysregulated mitophagy flux rather than uniformly activating mitophagy in all settings [33].

Mitochondrial biogenesis constitutes the third arm of MQC [19]. The principal molecular mechanisms through which melatonin regulates mitochondrial quality control—including redox regulation, mitochondrial dynamics, mitophagy, biogenesis, and mitochondrial–inflammatory signaling—are summarized in Table 1. Melatonin has been reported to promote mitochondrial biogenesis through pathways involving MT1, SIRT1, and PGC-1α, with downstream upregulation of nuclear respiratory factor 1 (NRF1) and mitochondrial transcription factor A (TFAM) [45]. The upstream mechanism linking melatonin to SIRT1 activation is likely context-dependent [9]. Receptor-mediated signaling through MT1/MT2 may activate intracellular pathways that converge on SIRT1, whereas receptor-independent mitochondrial actions may preserve NAD+-dependent sirtuin activity by reducing oxidative stress [45]. Activated SIRT1 promotes deacetylation and activation of PGC-1α, which then translocates to the nucleus and coactivates NRF1-dependent transcription of TFAM [45]. TFAM subsequently supports mtDNA transcription and replication, contributing to mitochondrial biogenesis [43]. However, the relative contribution of receptor-dependent versus receptor-independent SIRT1 activation remains incompletely defined in human reproductive tissues [4,16]. By coupling mitophagy-driven turnover with biogenesis-driven replenishment, melatonin may help preserve mitochondrial quantity and functional quality, a prerequisite for high-energy processes such as oocyte maturation and early embryogenesis [11,46].

Collectively, the available evidence supports a model in which melatonin acts as an integrative modulator of mitochondrial quality control rather than merely as a direct free radical scavenger.

### 3.4. Immunometabolic Convergence: Mitochondria as the Nexus of Inflammation Control

Mitochondria are increasingly recognized as signaling platforms linking cellular metabolism and innate immune activation [23]. Excessive mitochondrial ROS can promote mtDNA release through oxidative mtDNA damage, destabilization of mitochondrial membranes, opening of the mitochondrial permeability transition pore (mPTP), and mitochondrial outer membrane permeabilization [43]. Once released into the cytosol, mtDNA functions as a mitochondrial damage-associated molecular pattern (DAMP) and can amplify inflammatory signaling [18]. Importantly, NF-κB and the NLRP3 inflammasome are not produced within the mitochondrial matrix [47]. Rather, mitochondrial injury provides upstream danger signals, including mtROS and cytosolic mtDNA, that activate cytosolic and nuclear inflammatory pathways [47]. NF-κB activation occurs through cytoplasmic signaling followed by nuclear translocation to induce transcription of inflammatory genes, whereas NLRP3 inflammasome assembly occurs in the cytosol, often in proximity to damaged mitochondria or mitochondria-associated membranes [43,47]. Thus, mitochondria act as upstream inflammatory signaling platforms rather than the site of NF-κB or NLRP3 production [47]. By helping maintain mitochondrial integrity through MQC-related mechanisms, melatonin may reduce mtROS generation and cytosolic mtDNA release, thereby attenuating NF-κB activation, NLRP3 inflammasome assembly, and the maturation and release of downstream inflammatory cytokines such as interleukin-1β and interleukin-18 [47,48].

**Table 1:** Molecular mechanisms of melatonin regulating mitochondrial quality control.

MQC Component	Key Molecular Targets	Mechanism of Action	Functional Outcome	Key References
ROS regulation	ETC Complex I, Complex III	Direct scavenging of O_2_•^−^, •OH, and peroxynitrite-related oxidants; antioxidant metabolite cascade via C3-OHM, AFMK, and AMK; reduction in ETC electron leakage	Reduced oxidative damage to mitochondrial proteins, lipids, and mtDNA; improved redox stability	[9,37]
Mitochondrial dynamics	Drp1, OPA1, Mfn1/2	Inhibition of excessive mitochondrial fission and preservation of mitochondrial fusion	Maintenance of mitochondrial network stability and ATP production	[22,49]
Mitophagy	PINK1, Parkin	Modulation of PINK1/Parkin-mediated recognition of damaged mitochondria, ubiquitination of outer mitochondrial membrane proteins, and autophagosome–lysosome clearance	Improved clearance of damaged mitochondria and normalization of dysregulated mitophagy flux	[25,44]
Biogenesis	SIRT1, PGC-1α, NRF1, TFAM	Activates SIRT1–PGC-1α signaling leading to mitochondrial biogenesis	Restoration of mitochondrial population and metabolic capacity	[19,45]
Mitochondrial–inflammatory signaling	mtROS, mtDNA, NLRP3 inflammasome, NF-κB	Reduction in mitochondrial ROS and prevention of mtDNA release, leading to suppression of inflammasome activation	Attenuation of inflammatory signaling and preservation of tissue homeostasis	[20,23,50]

Abbreviations: MQC, mitochondrial quality control; ETC, electron transport chain; ROS, reactive oxygen species; mtROS, mitochondrial reactive oxygen species; Drp1, dynamin-related protein 1; OPA1, optic atrophy 1; Mfn1/2, mitofusin 1 and mitofusin 2; PINK1, PTEN-induced kinase 1; Parkin, E3 ubiquitin-protein ligase Parkin; SIRT1, sirtuin 1; PGC-1α, peroxisome proliferator-activated receptor gamma coactivator-1 alpha; NRF1, nuclear respiratory factor 1; TFAM, mitochondrial transcription factor A; mtDNA, mitochondrial DNA; NLRP3, NOD-like receptor family pyrin domain-containing 3; NF-κB, nuclear factor kappa-light-chain-enhancer of activated B cells.

In immune and reproductive tissues, endogenous melatonin synthesis further amplifies this immunometabolic regulation, enabling local control of mitochondrial redox balance during inflammatory activation [18,51]. This mitochondria-centered immunomodulation provides a mechanistic bridge linking melatonin’s molecular actions to attenuation of cytokine-driven pathology, including the inflammatory milieu characteristic of endometriosis and systemic hyperinflammatory states [14,18].

**Figure 2:**
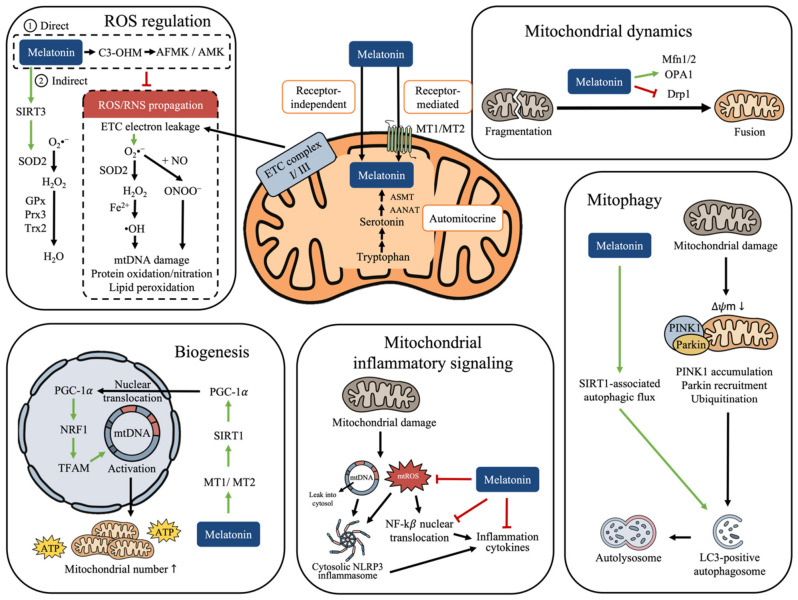
**Subcellular molecular pathways of melatonin-mediated mitochondrial quality control (MQC).** The schematic summarizes proposed molecular pathways through which melatonin may regulate five components of mitochondrial homeostasis. Green arrows denote activation or upregulation; red flat-ended lines denote inhibition or suppression; solid black arrows indicate molecular translocation, metabolic progression, or physiological state transitions. **Central Hub (Melatonin Pool):** Melatonin may reach or influence mitochondria through receptor-independent membrane diffusion, receptor-mediated signaling via MT1/MT2 receptors, and local intramitochondrial synthesis. Tryptophan is converted to serotonin, which is then sequentially catalyzed by matrix-localized AANAT and ASMT enzymes into melatonin (the Automitocrine loop). Melatonin has been proposed to interact with the electron transport chain, particularly complexes I and III, thereby limiting electron leakage and ROS generation. **ROS Regulation:** Electron leakage from ETC complexes I and III generates superoxide anion (O_2_•^−^), which is converted by SOD2 into hydrogen peroxide (H_2_O_2_). H_2_O_2_ may further generate hydroxyl radicals (•OH) through Fe^2+^-dependent Fenton-type chemistry, whereas O_2_•^−^ can react with nitric oxide (NO) to form peroxynitrite (ONOO^−^). These ROS/RNS contribute to mtDNA damage, protein oxidation or nitration, and lipid peroxidation. Melatonin attenuates this cascade through two complementary mechanisms: (1) a direct radical-scavenging pathway, in which melatonin is oxidatively converted to cyclic 3-hydroxymelatonin (C3-OHM), AFMK, and AMK, thereby extending antioxidant activity; and (2) an indirect enzymatic pathway involving SIRT3/SOD2 activation, which promotes superoxide dismutation and downstream detoxification of H_2_O_2_. Red blunt-ended lines indicate attenuation of ROS/RNS propagation and downstream mitochondrial oxidative/nitrative damage. **Mitochondrial Dynamics:** Melatonin has been reported to attenuate disease-associated mitochondrial fragmentation by reducing excessive Drp1-mediated fission and simultaneously upregulating the fusion proteins Mfn1/2 and OPA1, promoting a healthy, fused mitochondrial network. **Biogenesis:** Melatonin may promote mitochondrial biogenesis through receptor-mediated MT1/MT2 signaling and SIRT1-associated activation of PGC-1α. Activated PGC-1α translocates to the nucleus, where it coactivates NRF1-dependent transcriptional programs. NRF1 upregulates TFAM, which subsequently supports mtDNA transcription and replication, leading to increased mitochondrial biogenesis and ATP-generating capacity. This MT1/SIRT1/PGC-1α–NRF1–TFAM axis is presented as a proposed pathway that remains to be further validated in human reproductive tissues. **Mitochondrial Inflammatory Signaling:** Following mitochondrial damage, melatonin may reduce the release of mitochondrial damage-associated signals, including mtDNA and mtROS. Consequently, melatonin inhibits the activation of the NLRP3 inflammasome and suppresses the NF-κB pathway, thereby preventing the release of inflammatory cytokines. **Mitophagy:** Mitochondrial damage and loss of mitochondrial membrane potential (ΔΨm) promote PINK1 accumulation and Parkin recruitment. Parkin-mediated ubiquitination marks damaged mitochondria for recognition by LC3-positive autophagosomal membranes, followed by autophagosome–lysosome fusion and autolysosomal degradation. Melatonin may support this process by reducing mitochondrial ROS burden and by promoting SIRT1-associated autophagic flux, thereby normalizing dysregulated mitophagy flux rather than acting as a universal mitophagy activator. Abbreviations: MQC, mitochondrial quality control; ROS, reactive oxygen species; RNS, reactive nitrogen species; ETC, electron transport chain; O_2_•^−^, superoxide anion; H_2_O_2_, hydrogen peroxide; •OH, hydroxyl radical; NO, nitric oxide; ONOO^−^, peroxynitrite; Fe^2+^, ferrous iron; C3-OHM, cyclic 3-hydroxymelatonin; AFMK, N1-acetyl-N2-formyl-5-methoxykynuramine; AMK, N1-acetyl-5-methoxykynuramine; SIRT3, sirtuin 3; SOD2, superoxide dismutase 2; GPx, glutathione peroxidase; Prx3, peroxiredoxin 3; Trx2, thioredoxin 2; mtDNA, mitochondrial DNA; MT1/2, melatonin receptor 1 and melatonin receptor 2; AANAT, arylalkylamine N-acetyltransferase; ASMT, acetylserotonin O-methyltransferase; Mfn1/2, mitofusin 1 and mitofusin 2; OPA1, optic atrophy 1; Drp1, dynamin-related protein 1; PINK1, PTEN-induced kinase 1; Parkin, E3 ubiquitin-protein ligase Parkin; LC3, microtubule-associated protein 1 light chain 3; SIRT1, sirtuin 1; PGC-1α, peroxisome proliferator-activated receptor gamma coactivator-1 alpha; NRF1, nuclear respiratory factor 1; TFAM, mitochondrial transcription factor A; ATP, adenosine triphosphate; NLRP3, nucleotide-binding oligomerization domain-like receptor family pyrin domain-containing 3; NF-κB, nuclear factor kappa-light-chain-enhancer of activated B cells; ΔΨm, mitochondrial membrane potential.

Collectively, available evidence supports a model in which melatonin functions as an integrative modulator of mitochondrial quality control rather than merely as a direct free radical scavenger [19]. Through coordinated modulation of mitochondrial redox status, dynamics, mitophagy, biogenesis, and immunometabolic signaling, melatonin preserves mitochondrial homeostasis across diverse cell types [33]. This MQC-centered molecular architecture provides a unifying explanatory framework that connects melatonin’s subcellular actions to its broad physiological and clinical effects, setting the stage for its application in reproductive and inflammatory disorders. A comprehensive overview of these interconnected molecular processes, including both receptor-dependent and direct mitochondrial actions, is presented in Figure 2.

## 4. Clinical Evidence: Translational Implications of Melatonin-Mediated Mitochondrial Regulation

Before discussing specific clinical contexts, it is important to distinguish between mechanistic evidence and clinical outcome data. Most molecular evidence supporting melatonin-mediated mitochondrial quality control (MQC) regulation derives from in vitro systems, animal models, or isolated reproductive cells, whereas most human studies have evaluated macroscopic reproductive outcomes such as oocyte maturation, embryo quality, pregnancy rate, pain scores, or circulating biochemical markers. Direct evidence linking melatonin supplementation to MQC engagement in human follicular fluid, granulosa cells, or endometrial tissue remains limited [28,52]. Therefore, the following sections interpret melatonin as a promising adjunctive strategy, whose mechanistic rationale is supported mainly by preclinical data, while acknowledging that definitive human mitochondrial biomarker validation is still required [17]. Representative preclinical, observational, randomized, and meta-analytic evidence evaluating melatonin in reproductive disorders is summarized in Table 2, highlighting heterogeneity in study design, dosing strategies, outcome measures, and the limited use of direct mitochondrial biomarkers across clinical contexts.

As shown in Table 2, melatonin has been investigated across diverse reproductive conditions, including assisted reproductive technology, endometriosis, and polycystic ovary syndrome. The following sections discuss these contexts in detail from a mitochondria-centered perspective.

### 4.1. Melatonin in Assisted Reproductive Technology and Infertility

Assisted reproductive technology (ART) provides a clinically relevant context in which mitochondrial function plays a critical role in determining oocyte competence and early embryonic development [4]. Oocytes and early embryos are among the most mitochondria-dependent cells in human physiology, relying almost exclusively on oxidative phosphorylation to sustain meiotic progression, fertilization, and early cleavage [4,57]. Accumulating evidence indicates that age-related infertility and poor ART outcomes are closely associated with mitochondrial dysfunction and impaired mitochondrial quality control (MQC), manifested by excessive mitochondrial fragmentation, reduced ATP production, increased mitochondrial DNA mutations, and defective mitophagy [17,58]. Evidence in ART should be interpreted according to study type. Experimental studies provide mechanistic support for melatonin-mediated improvement of mitochondrial membrane potential, redox balance, and mitochondrial turnover in oocytes and granulosa cells [17,51]. In contrast, human studies have primarily reported associations between follicular melatonin levels and reproductive outcomes or evaluated supplementation effects on oocyte and embryo parameters [53]. Few clinical studies have directly measured MQC biomarkers in human follicular fluid, granulosa cells, or oocytes [17].

Within this context, melatonin has been proposed as a potential adjunct in ART due to its capacity to modulate mitochondrial function at multiple regulatory levels [59]. In follicular fluid, melatonin concentrations are markedly higher than in systemic circulation, suggesting a local requirement for mitochondrial protection during oocyte maturation [60,61]. Experimental studies suggest that melatonin can improve mitochondrial function in oocytes and granulosa cells, whereas human clinical studies have reported variable improvements in oocyte maturation, fertilization, or embryo-quality endpoints. These observations are consistent with, but do not directly prove, MQC engagement in human ART settings [59]. In preclinical models, these effects have been associated with improved mitochondrial membrane potential, more balanced mitochondrial dynamics, and enhanced clearance of damaged mitochondria. However, comparable mitochondrial endpoints are rarely incorporated into human ART trials [25,58].

Mechanistically, melatonin has been reported to preserve mitochondrial quality within the ovarian microenvironment through coordinated regulation of mitochondrial dynamics and turnover [58]. Experimental studies have suggested that melatonin may suppress excessive Drp1-mediated mitochondrial fission while supporting fusion-dependent maintenance of cristae structure [58,62]. In granulosa cell models, melatonin has been shown to activate SIRT1-dependent mitophagy pathways, facilitating selective elimination of dysfunctional mitochondria [25]. This dual action—mitochondrial preservation coupled with quality-controlled turnover—provides a biologically plausible mechanistic framework that may partially account for improvements reported in several experimental and clinical ART studies [56]. However, variability in trial design, patient selection, and outcome definitions warrants cautious interpretation.

Framing melatonin’s potential effects in ART within a mitochondrial quality control framework rather than an isolated antioxidant model may help explain several inconsistencies reported in earlier studies [33,63].

Despite encouraging findings, substantial heterogeneity exists across clinical trials evaluating melatonin in ART [6]. Several clinical studies evaluating melatonin supplementation in ART have administered oral doses of melatonin approximately 3 mg daily, although higher-dose protocols have also been reported, typically initiated several weeks prior to oocyte retrieval [4,6]. A smaller number of studies have explored higher daily doses (6–10 mg), while others have combined melatonin with adjunctive antioxidants such as vitamin E or myo-inositol, complicating mechanistic attribution [64].

Notably, few trials have incorporated pharmacokinetic assessments or measured follicular fluid melatonin concentrations to confirm tissue-level exposure [52]. Given that intramitochondrial melatonin concentrations are reported to far exceed circulating plasma levels, reliance on systemic dosing parameters alone may inadequately reflect target engagement within ovarian mitochondria [30,65]. This disconnect between systemic dosing and subcellular pharmacodynamics likely contributes to variability in clinical outcomes [31]. Taken together, melatonin remains a promising adjunctive strategy in ART, but its clinical utility should be interpreted cautiously until trials incorporate standardized dosing, tissue-level exposure assessment, and MQC-related biomarkers.

### 4.2. Melatonin in Endometriosis: Mitochondrial Dysfunction as a Therapeutic Target

Endometriosis is a chronic inflammatory disorder in which mitochondrial dysfunction has been proposed to contribute to lesion survival, oxidative stress, and inflammatory persistence [52,66]. Ectopic endometrial lesions are characterized by sustained oxidative stress, altered mitochondrial dynamics, impaired mitophagy, and metabolic reprogramming that collectively promote lesion survival and inflammatory persistence [13,14]. In this setting, excessive mitochondrial ROS generation and mitochondrial DNA release act as potent danger signals, driving activation of NF-κB signaling and assembly of the NLRP3 inflammasome [14,67].

**Figure 3:**
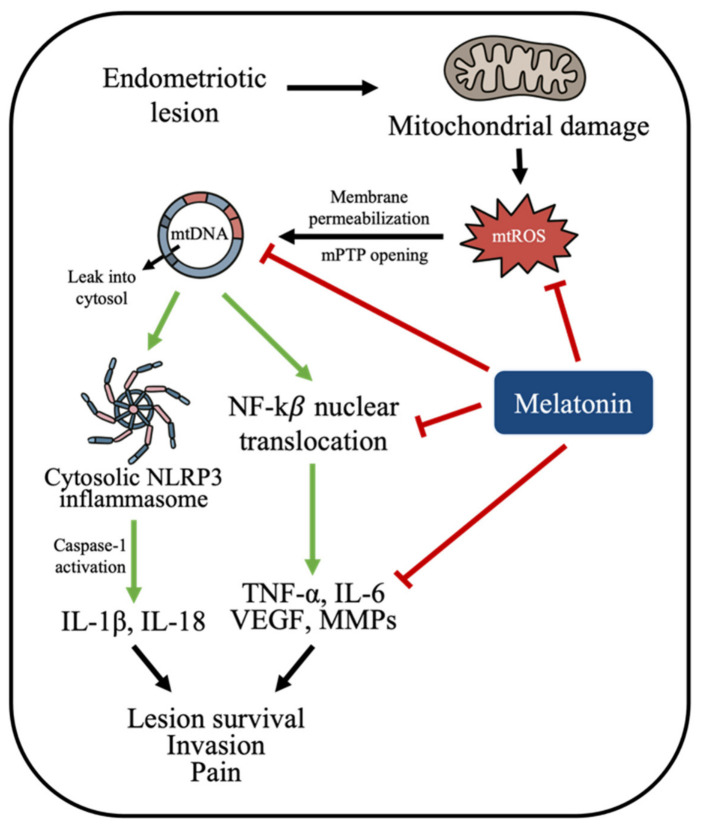
Proposed mitochondrial inflammatory signaling in endometriosis and its modulation by melatonin. Green arrow: promote; Red line: inhibit. In endometriotic lesions, oxidative stress and mitochondrial injury promote mitochondrial reactive oxygen species (mtROS) generation. Excessive mtROS may induce mitochondrial membrane permeabilization and mitochondrial permeability transition pore (mPTP) opening, leading to mitochondrial DNA (mtDNA) release into the cytosol. Cytosolic mtDNA functions as a mitochondrial damage-associated molecular pattern (DAMP) and, together with mtROS, contributes to activation of inflammatory signaling pathways. These signals promote nuclear factor kappa B (NF-κB) nuclear translocation and cytosolic nucleotide-binding oligomerization domain-like receptor family pyrin domain-containing 3 (NLRP3) inflammasome assembly. NLRP3 activation leads to caspase-1 activation and maturation of interleukin-1β (IL-1β) and interleukin-18 (IL-18), whereas NF-κB signaling promotes transcriptional activation of inflammatory mediators such as tumor necrosis factor-alpha (TNF-α), interleukin-6 (IL-6), vascular endothelial growth factor (VEGF), and matrix metalloproteinases (MMPs). Together, these inflammatory and tissue-remodeling mediators may contribute to lesion survival, invasion, and pain-related symptoms. Melatonin may attenuate this mitochondrial-inflammatory axis by reducing mtROS generation, limiting mtDNA release, and suppressing NF-κB/NLRP3-mediated inflammatory signaling. Black arrows indicate disease-associated signaling progression; red blunt-ended lines indicate melatonin-mediated inhibition. Abbreviations: mtROS, mitochondrial reactive oxygen species; mtDNA, mitochondrial DNA; mPTP, mitochondrial permeability transition pore; DAMP, damage-associated molecular pattern; NF-κB, nuclear factor kappa-light-chain-enhancer of activated B cells; NLRP3, nucleotide-binding oligomerization domain-like receptor family pyrin domain-containing 3; IL, interleukin; TNF-α, tumor necrosis factor-alpha; VEGF, vascular endothelial growth factor; MMPs, matrix metalloproteinases.

Melatonin’s therapeutic effects in endometriosis can be mechanistically interpreted through its capacity to restore mitochondrial quality control within ectopic and eutopic endometrial tissues [13,25]. Preclinical models have reported that melatonin can reduce lesion size, suppress inflammatory cytokine production, and alleviate pain-related behaviors [14,68]. At the molecular level, these effects coincide with attenuation of mitochondrial ROS production, stabilization of mitochondrial membrane integrity, and suppression of inflammasome activation [67,69]. The evidence supporting this mitochondrial-inflammatory interpretation is strongest in preclinical models. Human clinical evidence remains more limited and has primarily focused on pain-related outcomes or inflammatory markers rather than direct measurement of mitochondrial ROS, mtDNA release, inflammasome activation, or MQC biomarkers in endometriotic lesions. The proposed mitochondrial-inflammatory sequence linking mitochondrial damage, mtROS generation, cytosolic mtDNA release, NF-κB activation, NLRP3 inflammasome assembly, and downstream inflammatory mediator production in endometriosis is summarized in Figure 3, together with the potential inhibitory effects of melatonin on this pathway.

By helping preserve mitochondrial integrity, melatonin may reduce the cytosolic leakage of mitochondrial danger-associated molecular patterns, thereby interrupting the feed-forward loop between mitochondrial dysfunction and chronic inflammation [67]. Moreover, regulation of mitochondrial dynamics and mitophagy limits the persistence of dysfunctional, ROS-generating mitochondria that otherwise sustain inflammatory signaling [58,70]. This immunometabolic convergence positions melatonin not merely as an anti-inflammatory agent but as a modulator of the mitochondrial-inflammatory axis underlying endometriosis pathophysiology [14].

Viewed through this framework, endometriosis can be considered a prototypical disease model in which mitochondrial dysfunction and impaired mitochondrial quality control contribute to disease progression and inflammatory pathology [14]. Melatonin’s capacity to restore mitochondrial quality control provides a plausible mechanistic explanation for its reported analgesic, anti-inflammatory, and lesion-suppressive effects observed in preclinical and early clinical studies [14,15,55]. Accordingly, melatonin should currently be viewed as a mechanistically plausible adjunctive approach for endometriosis rather than an established disease-modifying therapy.

### 4.3. Other Emerging Clinical Contexts: PCOS and Hyperinflammatory States

Polycystic ovary syndrome (PCOS) is increasingly conceptualized as a disorder of combined metabolic and reproductive dysfunction in which mitochondrial stress and impaired quality control contribute to endocrine–metabolic dysregulation [58,71]. Beyond clinical phenotypes such as anovulation and hyperandrogenism, cellular studies in granulosa cells and oocyte–cumulus complexes support a mechanistic model characterized by excessive mitochondrial ROS production, altered mitochondrial dynamics, and defective mitophagy—features consistent with MQC failure [17,58]. Within this framework, melatonin is of interest not simply as a sleep-related hormone, but as a mitochondria-permeable modulator capable of restoring mitochondrial turnover and redox stability in ovarian cells [16].

It is important to acknowledge that PCOS is a multifactorial endocrine-metabolic disorder characterized not only by mitochondrial dysfunction but also by hyperinsulinemia, hyperandrogenism, altered gonadotropin secretion, and chronic low-grade inflammation [72,73]. Therefore, melatonin-mediated modulation of MQC likely operates within a broader hormonal and metabolic network rather than functioning as an isolated therapeutic axis [16,72].

Clinically, melatonin supplementation in PCOS has been evaluated across heterogeneous trial designs, outcomes, and co-interventions [52,74]. However, these clinical outcomes should not be interpreted as direct evidence of MQC restoration, because most PCOS trials have not assessed mitochondrial membrane potential, mitophagic flux, mtDNA-related markers, or MQC regulator expression in ovarian tissues [52]. Importantly, the mechanistic plausibility of benefit in PCOS is strengthened by cell-based and animal data indicating that melatonin can modulate SIRT1-linked pathways and mitophagy programs in granulosa cells, potentially preserving mitochondrial competence in a microenvironment prone to oxidative and inflammatory stress [17,25]. From a mitochondria-centered perspective, PCOS represents a clinically relevant context in which melatonin-mediated modulation of MQC could, if confirmed in human ovarian tissues, contribute to improved follicular homeostasis, while also highlighting a key translational gap: many clinical trials do not incorporate mitochondrial biomarkers capable of confirming on-target MQC engagement [52].

Beyond reproductive disorders, hyperinflammatory states provide a second mechanistically coherent domain for melatonin, as excessive inflammation frequently converges on mitochondrial danger signaling [18]. Mitochondrial ROS and leakage of mitochondrial DNA (mtDNA) can amplify innate immune activation, including inflammasome assembly, thereby sustaining cytokine production and tissue injury [75]. In this setting, melatonin’s proposed benefit is best understood as upstream control of the mitochondrial–immune interface: by preserving mitochondrial integrity and limiting ROS-dependent danger-associated molecular patterns, melatonin may reduce inflammasome activation and downstream inflammatory cascades [67]. A focused review of melatonin’s effects on the NLRP3 inflammasome summarizes multiple signaling nodes through which melatonin can attenuate inflammasome activation across disease models [23]. While the strength of evidence in hyperinflammatory syndromes is context-dependent, this immunometabolic logic reinforces the central thesis of this review: melatonin’s pleiotropic clinical profile becomes coherent when interpreted through MQC and mitochondrial danger signaling rather than as a collection of disconnected pathway effects [19].

However, a direct causal link between melatonin supplementation and reproductive improvement remains insufficiently established in humans. Clinical investigations in PCOS have demonstrated similar variability. In PCOS populations, interventional studies most commonly employ 3–5 mg of oral melatonin daily for 8–12 weeks, often in combination with lifestyle modification or insulin-sensitizing agents such as metformin [52,59]. These variations in co-intervention and treatment duration limit direct cross-study comparison. Differences in baseline metabolic phenotype, duration of supplementation, and outcome definitions (e.g., ovulatory rate versus metabolic parameters) complicate cross-study comparison [16]. Importantly, none of the currently available trials have stratified participants based on mitochondrial functional status or measured MQC-related biomarkers in granulosa cells or follicular fluid [52]. As a result, it remains unclear whether observed clinical improvements reflect direct mitochondrial engagement or secondary systemic effects [12,52].

Collectively, the clinical contexts discussed in this section illustrate a consistent translational pattern [14]. In ART, endometriosis, PCOS, and selected inflammatory conditions, melatonin’s effects are most biologically interpretable when outcomes are mapped onto mitochondrial endpoints—redox pressure, membrane potential, organelle turnover, and the downstream consequences of mitochondrial damage signals [33,58,76]. This perspective may help explain why melatonin has been associated with improvements in some intermediate phenotypes, such as oocyte or embryo quality and inflammatory biomarkers, whereas hard clinical endpoints, including live birth or durable lesion regression, remain inconsistent across trials [52,68].

## 5. Discussion

### 5.1. Integrative Synthesis: MQC as the Convergent Platform of Melatonin Biology

The present review advances a mitochondria-centered interpretation of melatonin biology by positioning mitochondrial quality control (MQC) as a regulatory architecture through which melatonin’s pleiotropic actions converge [19,30]. Rather than viewing antioxidant activity, anti-inflammatory signaling, and metabolic modulation as independent properties, our analysis suggests that these effects are mechanistically integrated at the level of mitochondrial maintenance [9,77].

Across molecular studies, melatonin influences multiple interconnected arms of MQC, including redox buffering at the electron transport chain, regulation of mitochondrial dynamics (fusion–fission balance), activation of mitophagy, and stimulation of mitochondrial biogenesis [21,49]. These processes are not isolated events but function as coordinated components of a quality control network that determines organelle fitness and cellular resilience [78]. By stabilizing mitochondrial membrane potential, limiting mtDNA damage, and preventing accumulation of dysfunctional mitochondria, melatonin may help preserve bioenergetic capacity in tissues characterized by high metabolic demand, such as oocytes and proliferative endometrial cells [17,51].

When interpreted through this framework, melatonin’s reported effects in assisted reproductive technology (ART), endometriosis, and polycystic ovary syndrome (PCOS) can be interpreted more coherently [14]. In each context, mitochondrial dysfunction serves as a common pathogenic denominator, and MQC modulation provides a plausible mechanistic explanation for improvements in intermediate phenotypes such as oocyte competence, inflammatory marker reduction, or metabolic stabilization [58]. This integrative perspective reduces conceptual fragmentation and reconciles seemingly diverse biological outcomes within a shared mitochondrial framework [38].

### 5.2. Explaining Clinical Heterogeneity Through Mitochondrial Engagement

Despite promising mechanistic evidence, clinical outcomes of melatonin supplementation remain heterogeneous [68]. This variability has often been attributed to differences in study design, population characteristics, the amount or frequency of melatonin supplementation, or outcome definitions [52]. However, a mitochondria-centered framework offers an additional mechanistic explanation: clinical benefit may depend on the degree to which melatonin effectively engages mitochondrial quality control in a given biological context [33].

Most of the clinical trials have not incorporated direct measurements of mitochondrial function or MQC-related biomarkers [52]. As a result, it remains unclear whether negative or neutral reports of some trials reflect inadequate dosing, insufficient tissue penetration, or absence of mitochondrial dysfunction in the study population [33]. Without stratification based on mitochondrial function phenotype—such as baseline oxidative stress burden, mtDNA copy number, or mitophagic capacity—trial outcomes may obscure subgroup-specific responses [79].

This lack of mechanistic stratification likely contributes to inconsistent findings across ART and PCOS trials [56]. For example, individuals with pronounced oxidative or mitochondrial stress may obtain greater benefit from melatonin-mediated MQC modulation than those with relatively preserved mitochondrial function [9]. Thus, heterogeneity in clinical outcomes may reflect biological heterogeneity rather than therapeutic inefficacy. It should also be noted that clinical findings are not uniformly positive [14]. Some trials and systematic reviews have reported improvements in intermediate reproductive outcomes such as oocyte maturation, embryo quality, or selected biochemical markers, whereas effects on pregnancy rate, live birth, or long-term disease control remain inconsistent [64,68]. These discrepancies likely reflect differences in patient selection, dosing regimen, treatment duration, co-interventions, and outcome definitions [18]. Accordingly, current evidence supports melatonin as a promising adjunctive intervention rather than an established reproductive therapy [80].

### 5.3. Translational Limitations: Dosing and Biomarker Deficiency

A major translational limitation in current melatonin research is the lack of standardized dosing strategies and the limited integration of mitochondrial pharmacodynamic endpoints [52,80]. Dosing regimens exhibit substantial heterogeneity depending on the reproductive context while ART trials typically utilize 3 mg daily for short durations preceding oocyte retrieval [80,81], studies targeting endometriosis have tested higher sustained doses with longer duration, such as 10 mg daily for 8 weeks [55]. Nevertheless, plasma melatonin levels do not necessarily correlate with intramitochondrial concentrations, which are reported to be substantially higher due to preferential accumulation within mitochondria [38]. Consequently, reliance on systemic dosing parameters may inadequately capture target engagement at the subcellular level [31]. It remains uncertain whether current clinical doses consistently achieve optimal modulation of mitochondrial dynamics, mitophagy, or biogenesis within ovarian or endometrial tissues. Moreover, variations in timing of administration relative to circadian phase may further influence pharmacodynamic outcomes [3].

Equally important is the limited incorporation of mitochondrial-specific biomarkers in clinical studies [58,80]. Few reproductive clinical trials have incorporated endpoints of mitochondrial functions such as follicular-fluid oxidative markers, mtDNA-related measures, mitochondrial membrane potential, or expression of MQC-associated regulators such as Drp1, PINK1, Parkin, or PGC-1α [19,80]. Without such endpoints, it is difficult to establish causal linkage between melatonin supplementation and the MQC engagement extensively described in preclinical models in human tissues [19,30]. Integration of these biomarkers into future trial design would strengthen mechanistic inference and reduce translational ambiguity. To address these translational limitations, potential mitochondrial biomarkers that could be incorporated into future clinical trial designs are summarized in Table 3.

Incorporation of these biomarkers may enable direct assessment of mitochondrial target engagement and strengthen the mechanistic interpretation of clinical outcomes.

### 5.4. Distinguishing the MQC Framework from Conventional Antioxidant Models

A critical conceptual distinction emphasized in this review is that melatonin’s biological actions cannot be fully explained by simple free radical scavenging [38]. To further clarify the conceptual distinction between traditional antioxidant models and the MQC-centered framework proposed in this review, a comparative summary is presented in Table 4.

This comparison highlights that melatonin exerts its biological effects through higher-order regulation of mitochondrial quality control rather than solely through direct free radical scavenging. Traditional antioxidant models focus primarily on the neutralization of reactive species [12,36]. In contrast, the MQC framework encompasses organelle-level regulation, including dynamic remodeling, selective autophagic turnover, and coordinated biogenic replenishment [19].

This current system-level interpretation accounts for sustained cellular resilience beyond immediate redox buffering [19,30]. By influencing the post-translational regulation of Drp1, stabilizing PINK1/Parkin-mediated mitophagy, and activating SIRT1–PGC-1α–dependent biogenesis, melatonin modulates mitochondrial population quality rather than merely reducing oxidative burden [22,45,82]. Such regulation has broader implications for metabolic flexibility, inflammatory signaling thresholds, and reproductive competence [14,30].

Framing melatonin within this higher-order regulatory architecture differentiates the present review from prior literature that catalogs melatonin’s effects without integrating them into a unified mitochondrial systems model.

### 5.5. Biological and Experimental Limitations

Although the MQC-centered framework provides a coherent integrative model, several biological limitations must be acknowledged. A substantial proportion of mechanistic evidence derives from animal models or in vitro granulosa cell systems, whereas direct demonstration of MQC modulation in human ovarian or endometrial tissue remains relatively limited [17,83]. Moreover, the relative contribution of receptor-dependent versus receptor-independent mitochondrial effects has not been definitively quantified in vivo [7,32].

In addition, the extent to which orally administered melatonin achieves consistent intramitochondrial concentrations in reproductive tissues has not been systematically evaluated [19,30,80]. Without tissue-level pharmacodynamic assessment, mechanistic extrapolation from cellular systems to clinical contexts should be interpreted with caution.

## 6. Conclusions

Melatonin has emerged as a multifaceted regulator of mitochondrial function, extending far beyond its classical role as a free radical scavenger. In this review, we propose an MQC-centered framework in which melatonin may act as an integrative modulator of mitochondrial function, influencing five interrelated processes: ROS regulation, mitochondrial dynamics, mitophagy, biogenesis, and mitochondrial–inflammatory signaling.

By applying this MQC-centered perspective across key reproductive pathologies—including ART-associated oocyte aging, endometriosis, and polycystic ovary syndrome (PCOS)—a plausible mechanistic framework emerges. Rather than exerting isolated antioxidant effects, melatonin modulates mitochondrial integrity at the system level, thereby influencing cellular bioenergetics, inflammatory thresholds, and tissue homeostasis. This integrated regulation provides a biologically plausible explanation for its benefits reported in selected experimental and clinical studies, including improvements in intermediate reproductive and inflammatory outcomes.

Despite these promising findings, significant translational gaps remain. Current clinical studies of melatonin are limited by heterogeneous dosing strategies, insufficient consideration of circadian pharmacodynamics, and a lack of validated mitochondrial-specific biomarkers to confirm target engagement in human tissues. Furthermore, much of the mechanistic evidence is derived from in vitro or animal models, underscoring the need for direct validation in human reproductive systems.

Future research should prioritize the integration of MQC-relevant biomarkers, standardized dosing frameworks aligned with mitochondrial pharmacology, and well-designed clinical trials that bridge mechanistic insights with clinical endpoints. Such efforts will be essential to determine whether melatonin can be developed as a mitochondria-targeted adjunctive strategy in reproductive medicine.

In summary, framing melatonin within the MQC paradigm not only may help integrate its diverse biological effects but also provides a forward-looking roadmap for translational research and clinical application.

## Figures and Tables

**Table 2:** Clinical evidence of melatonin in reproductive disorders.

Study	Evidence Type	Study Design/Sample Size	Population or Model	Melatonin Dose/Duration	Mitochondrial or Oxidative Markers	Clinical Outcomes	References
Tong 2017	Observational human data	Retrospective cohort study/N = 61	IVF/ICSI patients	Endogenous (Not standardized)/Evaluated on oocyte retrieval day	Intrafollicular melatonin concentration measured as a marker of antioxidant capacity	Higher melatonin levels positively correlated with increased mature oocytes, fertilization rate, and blastocyst rate	[53]
Eryilmaz 2011	Randomized controlled trials	Randomized controlled trial/N = 60	Women undergoing IVF	3 mg/day/From day 3–5 of menstrual cycle until hCG injection	Not explicitly assessed (focused on clinical/embryological parameters)	Increased number of mature (MII) oocytes and improved top-quality embryos	[54]
Jamilian 2019	Randomized controlled trials	Double-blind, placebo-controlled RCT/N = 56	PCOS patients	10 mg/day (2 × 5 mg)/12 weeks	Reduced MDA and hs-CRP; increased TAC and GSH levels; downregulated IL-1 and TNF-α expression	Significantly reduced hirsutism and total testosterone	[52]
Schwertner 2013	Randomized controlled trials	Phase II, double-blind, placebo-controlled RCT/N = 40	Endometriosis patients	10 mg/day/8 weeks	Decreased serum BDNF (Brain-Derived Neurotrophic Factor) levels	Reduced daily pelvic pain (39.8%) and dysmenorrhea; lowered analgesic use by 80%; improved sleep quality	[55]
Hu 2020	Systematic review and meta-analysis	Systematic review and meta-analysis	ART studies	3–6 mg/day (Variable across trials)	Evaluated broad antioxidative benefits (e.g., reduced ROS in follicular fluid across multiple studies)	Significantly improved clinical pregnancy rate, oocyte maturation, and good quality embryos	[56]

Abbreviations: RCT, randomized controlled trial; IVF, in vitro fertilization; ICSI, intracytoplasmic sperm injection; PCOS, polycystic ovary syndrome; MII, metaphase II; MDA, malondialdehyde; hs-CRP, high-sensitivity C-reactive protein; BDNF, brain-derived neurotrophic factor; ART, assisted reproductive technology. Note: Most human studies summarized in this table evaluated clinical or biochemical outcomes rather than direct MQC-related endpoints. Therefore, mitochondrial mechanisms should be interpreted as biologically plausible explanations supported mainly by preclinical evidence, rather than as directly validated mechanisms in human reproductive tissues.

**Table 3:** Proposed mitochondrial biomarkers useful for future clinical trials.

Biomarker	Biological Meaning	Measurement Method	Clinical Relevance	Reference
mtDNA copy number	Indicator of mitochondrial abundance and integrity	qPCR in follicular fluid or granulosa cells	Reflects mitochondrial competence of oocytes	[79]
Mitochondrial membrane potential (ΔΨm)	Indicator of mitochondrial bioenergetic status	JC-1 staining or flow cytometry	Predicts oocyte developmental competence	[51]
ROS levels	Indicator of oxidative stress within mitochondria	DCFH-DA fluorescence assays	Reflects oxidative damage affecting fertility	[37]
Drp1 expression	Marker of mitochondrial fission activity	Western blot or immunofluorescence	Excessive fission linked to mitochondrial dysfunction	[22]
PINK1/Parkin expression	Indicators of mitophagy activation	qPCR/Western blot	Reflects mitochondrial quality control activation	[17]
PGC-1α expression	Regulator of mitochondrial biogenesis	qPCR/Western blot	Associated with mitochondrial renewal capacity	[45]

Abbreviations: mtDNA, mitochondrial DNA; ΔΨm, mitochondrial membrane potential; ROS, reactive oxygen species; Drp1, dynamin-related protein 1; PINK1, PTEN-induced kinase 1; Parkin, E3 ubiquitin-protein ligase Parkin; PGC-1α, peroxisome proliferator-activated receptor gamma coactivator-1 alpha; qPCR, quantitative polymerase chain reaction.

**Table 4:** Traditional antioxidant model vs. MQC-targeted framework.

Feature	Traditional Antioxidant Model	MQC-Targeted Framework (Proposed)
Primary mechanism	Direct scavenging of reactive oxygen species	Integrated regulation of mitochondrial quality control
Molecular targets	ROS molecules	Mitochondrial dynamics, mitophagy, and biogenesis pathways
Duration of action	Immediate and transient	Sustained through mitochondrial renewal
Biomarkers	Serum ROS, TAC, MDA	mtDNA copy number, Drp1, PINK1, mitochondrial membrane potential
Therapeutic objective	Reduce oxidative damage	Restore mitochondrial homeostasis and cellular resilience
Biological scope	Redox balance	Organelle-level metabolic regulation

Abbreviations: ROS, reactive oxygen species; TAC, total antioxidant capacity; MDA, malondialdehyde; mtDNA, mitochondrial DNA; Drp1, dynamin-related protein 1; PINK1, PTEN-induced kinase 1.

## Data Availability

No new data were created or analyzed in this study. Data sharing is not applicable to this article.
